# Physical examination tests of the shoulder: a systematic review and meta-analysis of diagnostic test performance

**DOI:** 10.1186/s12891-017-1400-0

**Published:** 2017-01-25

**Authors:** Sigmund Ø. Gismervik, Jon O. Drogset, Fredrik Granviken, Magne Rø, Gunnar Leivseth

**Affiliations:** 1 Department Physical Medicine and Rehabilitation, St.Olavs University Hospital, P.B. 3250 Sluppen, NO-7006 Trondheim, Norway; 2 Department of Public Health and General Practice, Norwegian University of Science and Technology, P.B. 8905 MTFS, 7491 Trondheim, Norway; 3 Institute of Neuromedicine, Faculty of Medicine, Norwegian University of Science and Technology, P.B 8905 MTFS, 7491 Trondheim, Norway; 4 Department of Orthopedic Surgery, Trondheim University Hospital, P.B. 3250 Sluppen, NO-7006 Trondheim, Norway; 5 Department of Clinical Medicine, Neuromuscular Diseases Research Group, UiT The Arctic University of Norway, N-9037 Tromsø, Norway; 6 Unicare Medical Rehabilitation Centre, Hokksund, Norway

**Keywords:** Shoulder, Shoulder pain, Physical examination, Clinical test, Diagnosis, SLAP (superior labral anterior posterior) lesion, Rotator cuff tear, Subacromial impingement, Systematic review, Meta-analysis

## Abstract

**Background:**

Physical examination tests of the shoulder (PETS) are clinical examination maneuvers designed to aid the assessment of shoulder complaints. Despite more than 180 PETS described in the literature, evidence of their validity and usefulness in diagnosing the shoulder is questioned.

**Methods:**

This meta-analysis aims to use diagnostic odds ratio (DOR) to evaluate how much PETS shift overall probability and to rank the test performance of single PETS in order to aid the clinician’s choice of which tests to use. This study adheres to the principles outlined in the Cochrane guidelines and the PRISMA statement. A fixed effect model was used to assess the overall diagnostic validity of PETS by pooling DOR for different PETS with similar biomechanical rationale when possible. Single PETS were assessed and ranked by DOR. Clinical performance was assessed by sensitivity, specificity, accuracy and likelihood ratio.

**Results:**

Six thousand nine-hundred abstracts and 202 full-text articles were assessed for eligibility; 20 articles were eligible and data from 11 articles could be included in the meta-analysis. All PETS for SLAP (superior labral anterior posterior) lesions pooled gave a DOR of 1.38 [1.13, 1.69]. The Supraspinatus test for any full thickness rotator cuff tear obtained the highest DOR of 9.24 (sensitivity was 0.74, specificity 0.77). Compression-Rotation test obtained the highest DOR (6.36) among single PETS for SLAP lesions (sensitivity 0.43, specificity 0.89) and Hawkins test obtained the highest DOR (2.86) for impingement syndrome (sensitivity 0.58, specificity 0.67). No single PETS showed superior clinical test performance.

**Conclusions:**

The clinical performance of single PETS is limited. However, when the different PETS for SLAP lesions were pooled, we found a statistical significant change in post-test probability indicating an overall statistical validity. We suggest that clinicians choose their PETS among those with the highest pooled DOR and to assess validity to their own specific clinical settings, review the inclusion criteria of the included primary studies. We further propose that future studies on the validity of PETS use randomized research designs rather than the accuracy design relying less on well-established gold standard reference tests and efficient treatment options.

**Electronic supplementary material:**

The online version of this article (doi:10.1186/s12891-017-1400-0) contains supplementary material, which is available to authorized users.

## Background

Physical examination tests of the shoulder (PETS) aim to reproduce specific symptoms and signs as an aid for clinicians in diagnosing the painful shoulder. However, more than 180 different single PETS have been described in the literature [1] making the choice of which tests to use challenging. In addition, confusion arises because different names are used for the same test (e.g. Supraspinatus test = Empty can test = Jobe’s test [2–4]). Also, different criteria of positivity have been used for the same test (e.g. both ‘weakness’ [2] and/or ‘pain’ [3] as criterion of positivity for the supraspinatus test). Last but not least, several of the single PETS have been used for several different shoulder diagnoses (e.g. Yergason’s test originally published as a test of biceps pathology [5] is also used as test of glenoid labral pathology [6]). At present, therefore, there is a need to clarify the basis for an evidence based approach [7].

The validity of PETS based on meta-analysis from studies in primary care settings is scarce due to primary studies of insufficient quality [8]. However, several meta-analyses on PETS have been published in the specialty care setting. In one of these, a meta-analysis limited to PETS for subacromial impingement syndrome [9], the diagnostic validity of ‘Hawkins’, ‘Supraspinatus’, ‘Drop arm’ and ‘Lift-off’ tests was concluded to be limited by low pooled likelihood ratio (LR), but that ‘Lift-off’ test could be used to rule in a subscapularis tear. A more recent meta-analysis on rotator cuff tear recommended the ‘External rotation lag sign’ and ‘Painful arc’ tests based on findings of the highest pooled estimate of positive likelihood ratio and smallest confidence interval [10]. However, there was no overlap between the two meta-analyses regarding the studies finally retained for statistical pooling. Two additional meta-analyses have been published on PETS for superior labral anterior posterior (SLAP) lesions. In the first., ‘Active compression’, ‘Anterior slide’, ‘Crank’ and ‘Speed’ tests were included in the meta-analysis and assessed by estimated receiver operating characteristic curves [11]. ‘Anterior slide’ was concluded to perform worse than the other three tests but there were otherwise no significant differences [11]. The second meta-analysis on SLAP lesions [12] assessed Compression-rotation, Crank, Relocation, Speed and Yergason tests by pooled positive likelihood ratios and concluded that only the Yergason test showed statistical significant validity based on a likelihood ratio of 2.29 [1.21, 4.33]. In the update [13] of the only previous meta-analysis that has analyzed single PETS for all shoulder diagnosis (not limited to a specific diagnosis) [14], the concusion was that no single PETS were pathognomonic for any specific diagnoses and that the performance of PETS in general was low.

Given that the previous meta-analysis included different PETS and came to different conclusions, there is still a lack of robust evidence guiding clinicians on which tests to use in clinical practice and there is a need to assess if they are useful at all. The previous meta-analyses [9–14] were all aimed to pool data for single PETS assuming they were based on different biomechanical rationales. Only one of them included PETS for all shoulder diagnoses. It is therefore reasonable to suggest a different approach to meta-analysis of PETS.

In this systematic review we want to initially include PETS for all shoulder diagnoses commonly seen in specialty shoulder clinics, but limit the meta-analysis to include only high quality primary studies with a low risk of bias. Furthermore, we will try to pool different PETS that are based on similar biomechanical rationales in order to evaluate the validity of PETS in general.

This meta-analysis aims to use diagnostic odds ratio (DOR) [15], to evaluate how much PETS shift overall probability and to rank the test performance of single PETS in order to aid the clinician’s choice of which tests to use.

## Methods

The protocol for this systematic review and meta-analysis adhered to the principles outlined in the handbooks of the Cochrane Collaboration [16], the Norwegian Knowledge Center for Health Services [17] and the preferred reporting items in systematic reviews and meta-analysis (PRISMA) statement [18].

### Search methods for identification and processing of the literature

The electronic database searches were done in two stages (up to 2011; 2010 to June 2016). First stage, the searches were made in Medline (1946-), Embase (1980-), SPORT Discus (1975-); AMED (1985-); PEDRO (1929-) and the Cochrane library/Central. The alteration of the original search strategies was performed in 2015 and was used for searching the databases from 2010 to 2016. This modified search strategy included additional database-specific search terms as well as relevant text-words. A modified version of the methodological filter for diagnostic accuracy studies was applied [19, 20] in all searches. Additional citation searching and tracking was performed using ISI, SCOPUS and Google Scholar. Relevant reference lists of guidelines and systematic reviews were also checked. For a detailed description of the search strategy for Ovid Medline and PubMed see Additional file 1.

The search results were imported into an electronic reference database (EndNote) for removal of duplicates and further processing. Abstracts and full text articles were thereafter screened by the eligibility criteria for the meta-analysis. All evaluations, including assessments of eligibility and quality, were done by pairs of authors. Consistent interpretation of the eligibility and quality assessment process was ensured in consensus meetings with all authors before the respective processes were started. If doubt or dissent arose within the pair, consensus was sought with the other authors.

### Eligibility criteria, quality assessment and meta-analysis

Full-text articles which met the initial eligibility criteria 1–8 (Table 2) were assessed for potential sources of bias by use of the original quality assessment tool for diagnostic accuracy studies (QUADAS) [21].

In line with recommendations [16, 21], the 14 original QUADAS questions were adapted and a scoring guide was developed specifically for this review (See Appendix 2 in Additional file 2 for a detailed description). 2 × 2 tables were constructed from articles which met all eligibility criteria (Table 1). In line with convention [22], 0.5 was automatically added to all cells of the 2 × 2 table if one cell was 0. A fixed effect model was used to calculate sensitivity, specificity, accuracy, likelihood ratios (LR+/−) and DOR from pooled 2 × 2 tables. Exclusion of potential outlier studies before final pooling of data was based on visual outlier appearance in a Funnel plot, measurement of Cooks distance and assessment of spectrum effects [23] including disease prevalence in primary studies deviating from the average for all PETS within each diagnostic category. The performance of Single PETS were assessed and ranked by pooled DOR for each test and likelihood ratios were calculated to assess clinically relevant shifts in probability. The diagnostic validity of PETS in general was assessed by pooling DOR for different PETS based on similar biomechanical rationale (only possible for SLAP lesions). DOR pooled for detection of SLAP lesions was visualized in a forest plot. Heterogeneity for data in the forest plot was assessed by chi-square and I-square. Both bivariate and hierarchical random effects modelling were planned as options in the case of pooling five or more studies with high levels of heterogeneity.

**Table 1:** Eligibility criteria for inclusion in the meta-analysis

Abstracts	1.	Single PETS were studied^a^
	2.	PETS were compared to a reference test
	3.	Living humans were studied (animal, cadaver and general anaesthetic studies were excluded)
	4.	Study was not merely about fractures, dislocations of joints or nerve dysfunction
	5.	Article was in English or Scandinavian languages
Full-text articles^a^	1–5.	Same as above
	6.	The study included at least 20 patients
	7.	Sensitivity or specificity was reported or possible to discern for at least one PETS
	8.	The reference test was plausible (Supplement) for the condition studied
	9.	Risk of bias was acceptable, ie. patient selection criteria were clearly described (QUADAS question 2) and at least 8 of the 14 QUADAS items were scored “yes”
Requirement for pooling of data	10.	Construction of 2 × 2 contingency tables was possible and at least 2 studies reported PETS that were conducted and interpreted in the same ways

PETS-physical examination test(s) of the shoulder, QUADAS-quality assessment tool for diagnostic accuracy studies, 1 Articles that met criteria 1–8 were assessed with QUADAS.

^a^Studies that reported test characteristics for several single tests or combinations were also included as long as data on test performance for at least one single test was provided

## Results

### Articles and PETS included in the meta-analysis

**Fig. 1:**
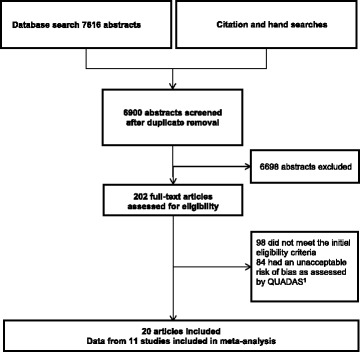
The flow of the search and selection process in this systematic review and meta-analysis of physical examination tests of the shoulder. ^1^QUADAS was scored for the all the articles that met the initial eligibility criteria. QUADAS-quality assessment tool for diagnostic accuracy studies

The flow of the search and selection process is presented in Fig. 1.

**Fig. 2:**
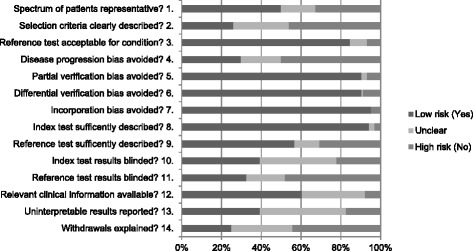
Risk of bias in the 104 articles assessed by QUADAS

From the 6900 abstracts and 202 full-text articles assessed for eligibility, 20 articles [2, 3, 6, 24–40] were found to have an acceptable risk of bias after QUADAS scoring (Fig. 2, Additional files 3, 4, and 5).

**Fig. 3:**
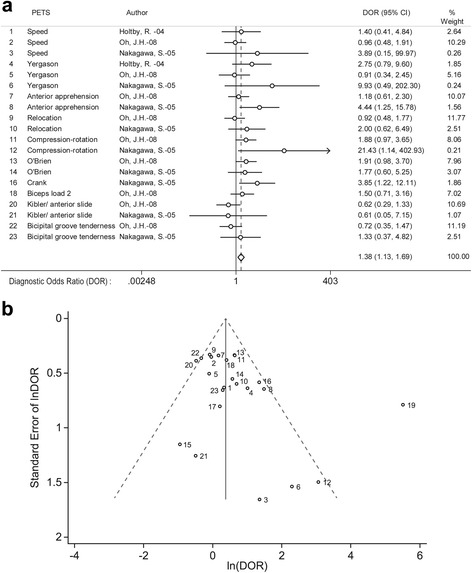
**a** Evidence for validity of PETS in diagnosing SLAP lesions. The diamond represents a pooled DOR of 1.38 with a 95% confidence interval of [1.13, 1.69]. The Forrest plot also visualizes that the variation in performance between the presumably different PETS was low. Heterogeneity chi-squared was 26.6 (d.f. = 19), *p* = 0.12; I-squared (variation in DOR attributable to heterogeneity) was 28.5%. PETS-physical examination tests of the shoulder, DOR-diagnostic odds ratio. **b** Funnel plot of 2 × 2 tables constructed for SLAP lesions. Nos. 15, 17 and 19 were omitted in the meta-analysis due to outlier characteristics; i.e. visual outlier appearance (No. 19), Cooks distance (No. 19) and disease prevalences (for the 10 PETS) deviating from the average 46% (72% for Nos. 15 and 17 and 31% for No. 19). Assessment of spectrum effects showed that Nos. 19 (Biceps load II test, (Kim, S.H -01)) and Nos. 15 and 17 (the O’Brien and Crank test, (Myers, T.H -05)) had included a non-representative spectrum of patients; they had low average ages (30.6 years [No. 19] and 23.9 years [Nos. 15&17]) and for Nos. 15&17 only athletes younger than 50 were included. Ln(DOR)-natural logarithmic transformation of diagnostic odds ratio

All the PETS reported in the 20 articles are listed in Appendix 1 (Additional file 2, see also Additional file 5 for extracted raw-data). Data from 11 articles, where at least two articles had described and interpreted the same single PETS the same way, was available for meta-analysis (see Additional file 6). The meta-analysis included PETS from three shoulder diagnoses (10 for SLAP lesions, two for subacromial impingement syndrome and one for rotator cuff tear). Subsequent assessments of outlier characteristics led to excluding one of the PETS [30] from the meta-analysis (Fig. 3).

### Evidence of diagnostic validity of PETS

Only PETS for SLAP lesions could be assessed for overall validity by pooling several different PETS based on similar biomechanical rationales. The pooled DOR of the included PETS for SLAP lesions was 1.38 [1.13, 1.69]. Heterogeneity chi-squared was 26.6 (d.f. = 19), *p* = 0.12; I-squared (variation in DOR attributable to heterogeneity) was 28.5% (Fig. 3a). A summary of results for the single PETS included in the meta-analysis is presented in Table 2.

**Table 2:** Diagnostic measures of single PETS ranked by DOR

PETS category	Single PETS	No. of studies	Pooled^a^ results (95% CI)				Accuracy rank	Likelihood ratios^c^
			Sensitivity	Specificity	DOR	Accuracy^b^		positive/negative
SLAP	Compression-rotation	2	0.43 (0.31, 0.56)	0.89 (0.67, 0.97)	6.36 (1.41, 28.59)	0.68 (0.59, 0.77)	1	3.91/0.64
	Yergason	3	0.20 (0.13, 0.30)	0.92 (0.81, 0.97)	2.91 (0.94, 9.08)	0.59 (0.54, 0.65)	3	2.50/0.87
	Anterior apprehension	2	0.74 (0.61, 0.84)	0.45 (0.35, 0.55)	2.29 (1.12, 4.69)	0.51 (0.51, 0.66)	4	1.35/0.58
	Crank	2	0.46 (0.33, 0.60)	0.72 (0.54, 0.85)	2.18 (0.82, 5.78)	0.60 (0.49, 0.71)	2	1.64/0.75
	Speed	3	0.20 (0.11, 0.32)	0.88 (0.73, 0.95)	1.73 (0.53, 5.65)	0.57 (0.49, 0.64)	5	1.67/0.91
	Relocation	2	0.61 (0.48, 0.72)	0.47 (0.37, 0.57)	1.36 (0.69, 2.66)	0.53 (0.45, 0.61)	6	1.15/0.83
	O'Brien	3	0.66 (0.55, 0.75)	0.36 (0.21, 0.55)	1.10 (0.46, 2.60)	0.50 (0.39, 0,60)	9	1.03/0.94
	Bicipital groove tenderness	2	0.26 (0.17, 0.37)	0.74 (0.63, 0.82)	0.98 (0.47, 2.05)	0.52 (0.45, 0.59)	7	1.00/1.00
	Kibler/anterior slide	2	0.10 (0.04, 0.23)	0.85 (0.73, 0.93)	0.61 (0.17, 2.23)	0.51 (0.44, 0.57)	8	0.67/1.06
SIS	Hawkins-Kennedy	2	0.58 (0.50, 0.66)	0.67 (0.47, 0.83)	2.86 (1.14, 7.17)	0.60 (0.53, 0.68)	1	1.76/0.63
	Neer	2	0.59 (0.52, 0.67)	0.60 (0.40, 0.77)	2.17 (0.91, 5.19)	0.59 (0.52, 0.67)	2	1.48/0.68
RCT^d^	Any full thickness RCT	2	0.74 (0.39, 0.92)	0.77 (0.69, 0.83)	9.24 (1.99, 42.84)	0.76 (0.63, 0.88)	NA	3.22/0.63
	Supraspinatus full thickness only	2	0.60 (0.46, 0.72)	0.70 (0.61, 0.78)	3.50 (1.74, 7.02)	0.66 (0.58, 0.73)	NA	2.00/0.57
	Any RCT	3	0.60 (0.52, 0.68)	0.63 (0.55, 0.71)	2.63 (1.62, 4.27)	0.62 (0.56, 0.68)	NA	1.62/0.63

*PETS* physical examination tests of the shoulder, *DOR* diagnostic odds ratio, *SLAP* superior labrum anterior superior, *SIS* subacromial impingement, *RCT* rotator cuff tear

^a^Calculated by a fixed effect model

^b^Based on average prevalences: 0.456 for SLAP lesions tests, 0.767 for SIS tests and 0.422 for the RCT test

^c^Calculated from pooled sensitivity and specificity

^d^Pooling was only possible for the Supraspinatus test (= Empty can test = Jobe's test); Weakness indicates positive test

The Compression-Rotation test [41] obtained the highest pooled DOR among single PETS in the SLAP category: DOR = 6.36 [1.41, 28.59]; specificity 0.89 and sensitivity 0.43. The highest ranks by pooled DOR for single PETS within the remaining shoulder diagnoses analyzed were the Hawkins test [42] for subacromial impingement syndrome: DOR = 2.86 [1.14, 7.17]; specificity 0.67, sensitivity 0.58; and the Supraspinatus test [4] for diagnosing any full thickness rotator cuff tear. The Supraspinatus test obtained the highest DOR overall: DOR = 9.24 [1.99, 42.84]; sensitivity 0.74, specificity 0.77.

## Discussion

This meta-analysis found statistical evidence for diagnostic validity of PETS when different tests for SLAP lesions were pooled (DOR = 1.38). Among the single PETS included in the meta-analysis, the highest DOR (9.24) overall was obtained for the Supraspinatus test in diagnosing any full thickness rotator cuff tear. The Compression-Rotation test was ranked highest of the SLAP tests (DOR 6.36) and the Hawkins test (DOR 2.86) for subacromial impingement syndrome (See Table 2 for details). However, the high risk of bias in primary studies and the fact that single PETS were performed and interpreted in diverging ways, limited the number of single PETS available for meta-analysis.

What constitutes superior clinical performance of a clinical test? In line with previous findings [13], no single PETS in this meta-analysis showed superior diagnostic validity when pooled test performance was assessed. An ideal test should have the ability to discriminate between subjects with and without the condition in question, i.e. a concurrent high sensitivity and specificity is sought. LR and DOR both convey a measurement for this concurrency (LR + =sensitivity/1-specificity; LR- = 1-sensitivity/specificity and DOR = LR+/LR-) of which DOR is the most sensitive single indicator of test performance [15]. For instance, when sensitivity and specificity both rise above 0.91; LR+ rises above 10 and DOR rises above 100. When reaching perfect test performance DOR rises to infinity. Nevertheless, LR may be more intuitive to the clinician when assessing clinical performance. According to Jaeschke et. al. [43], LR ratios >10 (LR+) or <0.1 (LR-) are needed to generate clinically conclusive changes in probability and moderate shifts are generated by a LR+ of 5–10 or LR- of 0.1-0.2.

When Walton et al. [12] recommended the Yergason test for SLAP lesions this was based on a pooled LR+ of 2.29. We found a similar LR+ (2.50) for the Yergason test and a slightly higher LR+ (3.91) for the Compression-Rotation test. However, when ranked by DOR the Yergason test performed second to Compression-Rotation test in our results (Table 2). None of the pooled results for single PETS resulted in LR+ above the range of 2–5 representing a small shift in probability [43].

The original study of the validity of a single PETS tend to report much better performance than later less biased attempts to replicate results. Despite the high sensitivity and specificity reported in the first study on Biceps load II [30], outlier characteristics led to exclusion from our meta-analysis (Fig. 3b). This decision is supported by previous reports about extensive bias in original studies and is in line with the exclusion of the original study on the Active Compression Test in a previous meta-analysis [13].

The forest plot (Fig. 3) visualizes the variation in the estimated performance of presumably different PETS. As we see, the estimated performance tends to vary between studies more than between the different tests, with a possible exception for the anterior slide test which also was found inferior to other SLAP tests in a previous meta-analysis [11]. In PETS aimed to detect SLAP lesions, most are designed to manipulate the superior labrum by stressing the glenohumeral joint often in combination with pulling on the biceps tendon (e.g. the Yergasons test of O’Brian test). This could be one of the reasons that performances of different tests vary relatively little, but this cannot explain why the general validity of PETS is poor. However, pathoanatomical/biomechanical rationale that most PETS are based on have recently been debated. For example, in subacromial impingement syndrome, the rationale for PETS (e.g. Hawkins and Neer’s sign tests) is that the greater tuberosity is rotated up underneath the acromion to force pinching of the bursa and supraspinatus tendon to reproduce impingement pain. The evidence for this postulated biomechanical explanation for the pain elicited is lacking [44]. Moreover, the fact that the interplay between genetics and psychological factors predicts shoulder pain in experimental and postoperative settings [45] also challenges the idea of a sole biomechanical explanation of shoulder pain.

In some of the previous meta-analysis of PETS hierarchical statistical modeling has been used to estimate receiver operating curves [9, 13]. No optimal curves for any single PETS have been documented apart from one possible exception for the Lift-off test though there was great uncertainty in the estimated curve. Hierarchical and bivariate random effects modeling were attempted also in our review but were not found feasible due to a low number of articles with acceptable risk of bias included for each single PETS. As heterogeneity was insignificant, a fixed effect model was used.

Despite the meticulous procedure to ensure high-quality input with an acceptable risk of bias, 9 of the 20 studies identified as eligible could not be included in the meta-analysis. In some, this was due to significant errors in reconstructing 2 × 2 tables such as test performance reported in the text of the result section that differed from that reported in tables [24] and that labels of several tables had been switched [28]. Unfortunately, some of these results have been included in previous systematic reviews [13].

Due to low quality of primary studies and strict selection criteria, we were only able to pool data for PETS within three shoulder diagnoses (SLAP lesions, subacromial impingement syndrome and for different degrees of rotator cuff tears only the supraspinatus test). Since gold standard reference tests have not been established for all shoulder diagnoses (e.g. multidirectional instability [46]), the accuracy study design itself may also present a challenge for the complete review of PETS as the validity of some PETS cannot be compared to a gold standard reference test. This may partially explain why no single PETS for multidirectional instability and adhesive capsulitis or other glenohumeral pathologies could be included in this meta-analysis. However, these and other shoulder diagnoses should still be assessed by the clinician as part of the general clinical examination.

The lack of uniform diagnostic labeling used in randomized controlled trials has led Schellingerhout et al. [7] to argue for abolishing diagnostic labels in shoulder pain patients altogether. Hence, there is a need for a new approach in future research on the validity of PETS and shoulder diagnoses. The GRADE initiative [47] suggests that validity of different diagnostic subgrouping strategies should be evaluated in a randomized design providing direct comparison of effects on patient-important outcomes (e.g. pain and shoulder function) for different diagnostic strategies, rather than the indirect evidence provided by the accuracy design. We therefore suggest that future research on the validity of PETS consider using such a randomized design.

### Limitations and strengths

This study adhered to the state of the art methodology for systematic reviews and diagnostic meta-analysis. A broad scope without limitations to any specific shoulder diagnoses was chosen to strengthen the potential clinical applicability of results. In the meta-analysis, a clear description of inclusion criteria was made mandatory for primary studies to ensure that applicability in other clinical settings can be assessed for all studies included. The chosen QUADAS cutoff in this study was in line with that used in several previous reviews [14, 48] and particularly strong selection criteria were used for the meta-analysis to ensure inclusion of only high quality primary studies with a low risk of bias. However, with strong selection criteria, there is a risk that relevant primary studies were excluded from the meta-analysis and that this may have biased our conclusions. In addition the application of a QUADAS cutoff score has been advised against by its developers [49] and our choice may have induced a selection bias of primary studies. Also, due to the small number of primary studies available for pooling, hierarchical or bivariate random effects modeling were not feasible. However, since heterogeneity was low, a fixed effects approach could be used. A revised edition of the original QUADAS tool has been published [50]. Implementation was not possible in this review as QUADAS scoring had already started with the original tool. This was a meta-analysis of single PETS but in clinical practice a combination of tests is commonly used. Several of the included primary studies reported diagnostic performance when different tests were combined [3, 26, 34, 35, 37]. However, as test combinations differ, meaningful statistical pooling was not feasible and assessment of test combinations was beyond the specific scope of this meta-analysis. Another important limitation regarding conclusions and recommendations of this meta-analysis is the designated context of specialist care with high prevalence of shoulder pathology and co-morbidity. Care should be taken to assess applicability of results to any specific clinical context. To enable clinicians to assess transferability of primary research findings to their own specific spectrum of patients, we only included studies where inclusion criteria had been clearly described. The extraction of raw data from the included primary studies have been provided for clinicians own scrutiny (Additional file 5).

## Conclusions

The clinical performance of single PETS is limited. However, our evidence indicates statistical validity when the different PETS for SLAP lesions were pooled. We suggest that clinicians choose their PETS among those with the highest rank of pooled DOR (Compression rotation, Yergason, Anterior apprehension or Crank tests for SLAP lesions; Hawkins-Kennedy for subacromial impingement and the supraspinatus/empty can/Jobe’s test for full thickness rotator cuff tears). Furthermore, we recommend that the clinician assess the inclusion criteria in relevant primary studies to assess the validity for their own clinical setting. There is still a need for a new research approach to the evidence based shoulder examination. A new approach to the diagnostic labels in the shoulder has also been called for by Schellingerhout et al. [7]. We therefore propose that future studies on the validity of PETS use a randomized research design [47] in order to compare the validity of different diagnostic strategies related to their effect on patient-outcomes.

## Additional files

Additional file 1: Table S1. Detailed description of the literature search strategy. (XLS 31 kb)
Additional file 2: Contains: a) Overview of PETS in the 20 articles with low risk of bias. b) Adapted QUADAS assessment tool and scoring guide. c) Full initial eligibility criteria for abstracts and full text articles. (DOC 104 kb)
Additional file 3: Table S2. Quality scores for the 20 full text articles with acceptable risk of bias. (XLSX 17 kb)
Additional file 4: QUADAS score table (containing scores for all articles assessed). (XLS 2873 kb)
Additional file 5: Data-extraction from 20 articles with low risk of bias (raw-data). (XLS 109 kb)
Additional file 6: Data-extraction prepared for Meta-analysis (raw-data). (XLS 83 kb)

## Abbreviations

DOR: Diagnostic Odds Ratio
LR +/−: Likelihood ratio positive/negative
PETS: Physical Examination Test(s) of the Shoulder
PRISMA: Preferred Reporting Items in Systematic Reviews and Meta-Analysis
QUADAS: Quality Assessment of Diagnostic Accuracy Studies
SLAP: Superior Labral Anterior Posterior

## References

[1] McFarland EG. Examination of the Shoulder: The Complete Guide. Thieme; 2006. ISBN: 1588903710. https://www.amazon.com/Examination-Shoulder-Complete-Edward-McFarland/dp/1588903710.

[2] Holtby R, Razmjou H (2004). Validity of the supraspinatus test as a single clinical test in diagnosing patients with rotator cuff pathology. J Orthop Sports Phys Ther.

[3] Chew K, Pua YH, Chin J, Clarke M, Wong YS (2010). Clinical predictors for the diagnosis of supraspinatus pathology. Physiotherapy Singapore.

[4] Jobe FW, Moynes DR (1982). Delineation of diagnostic criteria and a rehabilitation program for rotator cuff injuries. Am J Sports Med.

[5] Yergason RM. Supination sign. J Bone Joint Surg Am. 1931;13:160–160.

[6] Holtby R, Razmjou H (2004). Accuracy of the Speed’s and Yergason’s tests in detecting biceps pathology and SLAP lesions: comparison with arthroscopic findings. Arthroscopy.

[7] Schellingerhout JM, Verhagen AP, Thomas S, Koes BW (2008). Lack of uniformity in diagnostic labeling of shoulder pain: time for a different approach. Man Ther.

[8] Hanchard NC, Lenza M, Handoll HH, Takwoingi Y (2013). Physical tests for shoulder impingements and local lesions of bursa, tendon or labrum that may accompany impingement. Cochrane Database Syst Rev.

[9] Alqunaee M, Galvin R, Fahey T (2012). Diagnostic accuracy of clinical tests for subacromial impingement syndrome: a systematic review and meta-analysis. Arch Phys Med Rehabil.

[10] Hermans J, Luime JJ, Meuffels DE, Reijman M, Simel DL, Bierma-Zeinstra SM (2013). Does this patient with shoulder pain have rotator cuff disease?: The Rational Clinical Examination systematic review. JAMA.

[11] Meserve BB, Cleland JA, Boucher TR (2009). A meta-analysis examining clinical test utility for assessing superior labral anterior posterior lesions. Am J Sports Med.

[12] Walton DM, Sadi J (2008). Identifying SLAP lesions: a meta-analysis of clinical tests and exercise in clinical reasoning. Phys Ther Sport.

[13] Hegedus EJ. Which physical examination tests provide clinicians with the most value when examining the shoulder? Update of a systematic review with meta-analysis of individual tests. Br J Sports Med. 2012. 10.1136/bjsports-2012-091066 22773322

[14] Hegedus EJ, Goode A, Campbell S, Morin A, Tamaddoni M, Moorman CT, Cook C (2008). Physical examination tests of the shoulder: a systematic review with meta-analysis of individual tests. Br J Sports Med.

[15] Glas AS, Lijmer JG, Prins MH, Bonsel GJ, Bossuyt PM (2003). The diagnostic odds ratio: a single indicator of test performance. J Clin Epidemiol.

[16] Cochrane-Collaboration. Handbook for DTA Reviews. In: *Book Handbook for DTA Reviews*. Cochrane collaboration; 2011. srdta.cochrane.org/handbook-dta-reviews.

[17] NOKC (2009). Handbook for Norwegian Knowledge Center for the Health Services. Book Handbook for Norwegian Knowledge Center for the Health Services.

[18] Liberati A, Altman DG, Tetzlaff J, Mulrow C, Gotzsche PC, Ioannidis JP, Clarke M, Devereaux PJ, Kleijnen J, Moher D (2009). The PRISMA statement for reporting systematic reviews and meta-analyses of studies that evaluate health care interventions: explanation and elaboration. PLoS Med.

[19] Haynes RB, Wilczynski NL (2004). Optimal search strategies for retrieving scientifically strong studies of diagnosis from Medline: analytical survey. BMJ.

[20] Beynon R, Leeflang MM, McDonald S, Eisinga A, Mitchell RL, Whiting P, Glanville JM. Search strategies to identify diagnostic accuracy studies in MEDLINE and EMBASE. Cochrane Database Syst Rev 2013:MR000022. 10.1002/14651858.MR000022.pub3 PMC7390022 24022476

[21] Whiting PF, Weswood ME, Rutjes AW, Reitsma JB, Bossuyt PN, Kleijnen J (2006). Evaluation of QUADAS, a tool for the quality assessment of diagnostic accuracy studies. BMC Med Res Methodol.

[22] Deville WL, Buntinx F, Bouter LM, Montori VM, de Vet HC, van der Windt DA, Bezemer PD (2002). Conducting systematic reviews of diagnostic studies: didactic guidelines. BMC Med Res Methodol.

[23] Mulherin SA, Miller WC (2002). Spectrum bias or spectrum effect? Subgroup variation in diagnostic test evaluation. Ann Intern Med.

[24] Ardic F, Kahraman Y, Kacar M, Kahraman MC, Findikoglu G, Yorgancioglu ZR (2006). Shoulder impingement syndrome: relationships between clinical, functional, and radiologic findings. Am J Phys Med Rehabil.

[25] Bak K, Sorensen AKB, Jorgensen U, Nygaard M, Krarup AL, Thune C, Sloth C, Pedersen ST (2010). The value of clinical tests in acute full-thickness tears of the supraspinatus tendon: does a subacromial lidocaine injection help in the clinical diagnosis? a prospective study. Arthroscopy.

[26] Fodor D, Poanta L, Felea I, Rednic S, Bolosiu H (2009). Shoulder impingement syndrome: correlations between clinical tests and ultrasonographic findings. Ortop Traumatol Rehabil.

[27] Hertel R, Ballmer FT, Lombert SM, Gerber C (1996). Lag signs in the diagnosis of rotator cuff rupture. J Shoulder Elbow Surg.

[28] Kim E, Jeong HJ, Lee KW, Song JS (2006). Interpreting positive signs of the supraspinatus test in screening for torn rotator cuff. Acta Med Okayama.

[29] Kim HA, Kim SH, Seo YI (2007). Ultrasonographic findings of painful shoulders and correlation between physical examination and ultrasonographic rotator cuff tear. Mod Rheumatol.

[30] Kim SH, Ha KI, Ahn JH, Choi HJ (2001). Biceps load test II: A clinical test for SLAP lesions of the shoulder. Arthroscopy.

[31] Miller CA, Forrester GA, Lewis JS (2008). The validity of the lag signs in diagnosing full-thickness tears of the rotator cuff: a preliminary investigation. Arch Phys Med Rehabil.

[32] Myers TH, Zemanovic JR, Andrews JR (2005). The resisted supination external rotation test: a new test for the diagnosis of superior labral anterior posterior lesions. Am J Sports Med.

[33] Nakagawa S, Yoneda M, Hayashida K, Obata M, Fukushima S, Miyazaki Y (2005). Forced shoulder abduction and elbow flexion test: a new simple clinical test to detect superior labral injury in the throwing shoulder. Arthroscopy.

[34] Oh JH, Kim JY, Kim WS, Gong HS, Lee JH (2008). The evaluation of various physical examinations for the diagnosis of type II superior labrum anterior and posterior lesion. Am J Sports Med.

[35] Park HB, Yokota A, Harpreet GS, El Rassi G, McFarland EG (2005). Diagnostic accuracy of clinical tests for the different degrees of subacromial impingement syndrome. J Bone Joint Surg Am.

[36] Razmjou H, Holtby R, Myhr T (2004). Pain provocative shoulder tests: reliability and validity of the impingement tests. Physiother Can.

[37] Walton J, Mahajan S, Paxinos A, Marshall J, Bryant C, Shnier R, Quinn R, Murrell GA (2004). Diagnostic values of tests for acromioclavicular joint pain. J Bone Joint Surg Am.

[38] Zaslav KR (2001). Internal rotation resistance strength test: a new diagnostic test to differentiate intra-articular pathology from outlet (Neer) impingement syndrome in the shoulder. J Shoulder Elbow Surg.

[39] Collin P, Treseder T, Denard PJ, Neyton L, Walch G, Ladermann A (2015). What is the best clinical test for assessment of the teres minor in massive rotator cuff tears?. Clin Orthop Relat Res.

[40] Toprak U, Ustuner E, Ozer D, Uyanik S, Baltaci G, Sakizlioglu SS, Karademir MA, Atay AO (2013). Palpation tests versus impingement tests in Neer stage I and II subacromial impingement syndrome. Knee Surg Sports Traumatol Arthrosc.

[41] Snyder SJ, Karzel RP, Del Pizzo W, Ferkel RD, Friedman MJ (1990). SLAP lesions of the shoulder. Arthroscopy.

[42] Hawkins RJ, Kennedy JC (1980). Impingement syndrome in athletes. Am J Sports Med.

[43] Jaeschke R, Guyatt GH, Sackett DL (1994). Users’ guides to the medical literature. III. How to use an article about a diagnostic test. B. What are the results and will they help me in caring for my patients? The Evidence-Based Medicine Working Group. JAMA.

[44] Papadonikolakis A, McKenna M, Warme W, Martin BI, Matsen FA (2011). Published evidence relevant to the diagnosis of impingement syndrome of the shoulder. J Bone Joint Surg Am.

[45] George SZ, Wallace MR, Wright TW, Moser MW, Greenfield WH, Sack BK, Herbstman DM, Fillingim RB (2008). Evidence for a biopsychosocial influence on shoulder pain: pain catastrophizing and catechol-O-methyltransferase (COMT) diplotype predict clinical pain ratings. Pain.

[46] Saccomanno MF, Fodale M, Capasso L, Cazzato G, Milano G (2013). Generalized joint laxity and multidirectional instability of the shoulder. Joints.

[47] Schunemann HJ, Oxman AD, Brozek J, Glasziou P, Jaeschke R, Vist GE, Williams JW, Kunz R, Craig J, Montori VM (2008). Grading quality of evidence and strength of recommendations for diagnostic tests and strategies. BMJ.

[48] Wright AA, Wassinger CA, Frank M, Michener LA, Hegedus EJ. Diagnostic accuracy of scapular physical examination tests for shoulder disorders: a systematic review. Br J Sports Med. 2012. 10.1136/bjsports-2012-091573 23080313

[49] Whiting P, Harbord R, Kleijnen J (2005). No role for quality scores in systematic reviews of diagnostic accuracy studies. BMC Med Res Methodol.

[50] Whiting PF, Rutjes AW, Westwood ME, Mallett S, Deeks JJ, Reitsma JB, Leeflang MM, Sterne JA, Bossuyt PM (2011). QUADAS-2: a revised tool for the quality assessment of diagnostic accuracy studies. Ann Intern Med.

