# Supplementary material for: Physical examination tests of the shoulder: a systematic review and meta-analysis of diagnostic test performance
Source: BMC Musculoskelet Disord. 2017 Jan 25;18:41. doi: 10.1186/s12891-017-1400-0 (PMC5267375; doi:10.1186/s12891-017-1400-0)
Supplement: Additional file 2: — Contains: a) Overview of PETS in the 20 articles with low risk of bias. b) Adapted QUADAS assessment tool and scoring guide. c) Full initial eligibility criteria for abstracts and full text articles. (DOC 104 kb) [file 12891_2017_1400_MOESM2_ESM.doc]

# Physical Examination Tests of the Shoulder (PETS)

A Systematic Review and Meta-Analysis of Test Performance

**Index of supplementary material:**

Page 2-4:

Appendix 1: Overview of PETS included in 20 articles with acceptable risk of bias

Page 5-11:

Appendix 2: Adapted quality assessment tool with scoring guide

Page 12:

Appendix 3: Initial eligibility criteria for inclusion of articles in quality assessment

**The original protocol for this systematic review was published online:**

[https://stolav.no/Fysmed/Protokoll-systematic%20review%20of%20shoulder%20tests_English_ver2.0.pdf](https://stolav.no/Fysmed/Protokoll-systematic review of shoulder tests_English_ver2.0.pdf)

| Appendix 1 Overview of PETS included in 20 articles with acceptable risk of bias; inclusion criteria were clearly described and QUADAS score was ≥ 8 | |
| --- | --- |
| **PETS** | **Authors** (diagnostic categories studied) |
| Neer | Ardic, F. -06^1^ (RCT)  Bak, K. -10^1^ (acute traumatic RCT or bursitis)  Chew, K. -10 (supraspinatus pathology)  Fodor, D. -09^1^ (SIS different stages)  Kim, H. A.-07^1^ (pathologies diagnosed by ultrasound)  Nakagawa, S. -05 (SLAP lesions)  Park, H. B. -05 (SIS / bursitis / RCT)  Razmjou, H. -04 (SIS)  Toprak, U. -12 (SIS stages I and II) |
| Hawkins-Kennedy | Ardic, F. -06^1^ (RCT)  Bak, K. -10^1^ (acute traumatic RCT or bursitis)  Chew, K. -10 (supraspinatus pathology)  Fodor, D. -09^1^ (SIS different stages)  Kim, H. A.-07^1^ (pathologies diagnosed by ultrasound)  Nakagawa, S. -05 (SLAP lesions)  Park, H. B. -05 (SIS / bursitis / RCT)  Razmjou, H. et al.-04 (SIS)  Toprak, U. -12 ((SIS stages I and II) |
| Speed | Ardic, F. -06^1^ (RCT)  Holtby, R. -04^2^ (biceps tendon and SLAP lesions)  Nakagawa, S. -05 (SLAP lesions)  Oh, J.H.-08 (SLAP type II lesion)  Park, H. B. -05 (SIS / bursitis / RCT) |
| Yergasons's test | Holtby, R. -04^2^ (Biceps tendon / SLAP lesions)  Kim, H. A.-07^1^ (pathologies diagnosed by ultrasound)  Nakagawa, S. -05 (SLAP lesions)  Oh, J.H.-08 (SLAP type II lesion) |
| Active range of motion | Ardic, F. -06^1^ (RCT)  Kim, H. A.-07^1^ (pathologies diagnosed by ultrasound) |
| Passive range of motion | Kim, H. A.-07^1^ (pathologies diagnosed by ultrasound) |
| External rotation strength | Fodor, D. -09^1^ (SIS different stages)  Park, H. B. -05 (SIS / bursitis / RCT) |
| Painful arc | Bak, K. -10^1^ (acute traumatic RCT or bursitis)  Chew, K. -10 (supraspinatus pathology)  Fodor, D. -09^1^ (SIS different stages)  Nakagawa, S. -05 (SLAP lesions)  Park, H. B. -05 (SIS / Bursitis / RCT) |
| ERLS-'External Rotation Lag Sign' for supraspinatus tears | Bak, K. -10^1^ (acute traumatic RCT or bursitis)  Collin, P. -15 (RCT m. teres minor)  Hertel, R.- 96 (RCT in 7 categories)  Miller, C. A. -08^1^ (full thickness RCT  Park, H. B. -05 (bursitis / RCT) |
| IDS-'Infraspinatus Drop Sign'  for infraspinatus tears | Bak, K. -10^1^ (acute traumatic RCT or bursitis)  Hertel, R.- 96 (RCT in 7 categories)  Miller, C. A. -08^1^ (full thickness RCT) |
| DAT-'Drop Arm Test'  for supraspinatus tears’ | Bak, K. -10^1^ (acute traumatic RCT or bursitis)  Chew, K. -10 (supraspinatus pathology)  Park, H. B. -05 (SIS / Bursitis / RCT) |
| DAT-'Drop Arm Test'  for teres minor tears’ | Collin, P. -15 (RCT m. teres minor) |
| IRLS-'Internal Rotation Lag Sign'  for subscapularis tears | Bak, K. -10^1^ (acute traumatic RCT or bursitis)  Hertel, R.- 96 (RCT in 7 categories)  Miller, C. A. -08^1^ (full thickness RCT) |
| Yocum’s test | Fodor, D. -09^2^ (SIS different stages) |
| Supraspinatus test  (= Jobe’s = Empty can test) | Bak-10^1^ (acute traumatic RCT or bursitis)  Chew, K. -10 (supraspinatus pathology)  Fodor, D. -09^1^ (SIS different stages)  Hertel, R.- 96 (RCT in 7 categories)  Holtby, R. -04^3^ (any RCT that required repair)  Kim, E. -06^1^ (supraspinatus tear)  Kim, H. A.-07^1^ (pathologies diagnosed by US)  Park, H. B. -05 (SIS / bursitis / RCT) |
| Full can test | Chew, K. -10 (supraspinatus pathology)  Kim, E. -06^1^ (supraspinatus tear) |
| Pattes test | Collin, P. -15 (RCT m. teres minor)  Fodor-09^1^ (SIS different stages)  Kim, H. A.-07^1^ (pathologies diagnosed by ultrasound) |
| Gerber lift of test | Fodor, D. -09^1^ (SIS different stages)  Hertel, R.- 96 (RCT in 7 categories)  Kim, H. A.-07^1^ (pathologies diagnosed by ultrasound) |
| Anterior Apprehension  (= Fulcrum test) | Nakagawa, S. -05 (SLAP lesions)  Oh, J.H.-08 (SLAP type II lesion) |
| Relocation | Nakagawa, S. -05 (SLAP lesions)  Oh, J.H.-08 (SLAP type II lesion) |
| Compression-Rotation | Nakagawa, S. -05 (SLAP lesions)  Oh, J.H.-08 (SLAP type II lesion) |
| Active Compression Test  (= O'Briens test) | Myers, T. H. -05 (SLAP lesions)  Nakagawa, S. -05 (SLAP lesions)  Oh, J.H.-08 (SLAP type II lesion)  Walton, J. -04 (AC-joint pain) |
| Biceps load II | Oh, J.H.-08 (SLAP type II lesion)  Kim, S. H. -01 (SLAP lesions) |
| Anterior Slide  (= Kibler’s test) | Nakagawa, S. -05 (SLAP lesions)  Oh, J.H.-08 (SLAP type II lesion) |
| Whipple | Oh, J.H.-08 (SLAP type II lesion) |
| Bicipital Groove tenderness | Nakagawa, S. -05 (SLAP lesions)  Oh, J.H.-08 (SLAP type II lesion) |
| Glenohumeral tenderness | Kim, H. A.-07^1^ (pathologies diagnosed by ultrasound) |
| Subacromial tenderness | Kim, H. A.-07^1^ (pathologies diagnosed by ultrasound) |
| Crank test | Myers, T. H. -05 (SLAP lesions)  Nakagawa, S. -05 (SLAP lesions) |
| Resisted Supination External Rotation Test | Myers, T. H. -05 (SLAP lesions) |
| Forced Shoulder Abduction and Elbow Flexion Test | Nakagawa, S. -05 (SLAP lesions) |
| Sulcus test | Nakagawa, S. -05 (SLAP lesions) |
| Ellman test (for impingement); clunk test, ABIS test-‘abduction inferior stability test’; posterior jerk test (for SLAP) | Nakagawa, S. -05 (SLAP lesions) |
| Cross body | Chew, K. -10 (supraspinatus pathology)  Park, H. B. -05 (SIS / bursitis / RCT) |
| Paxinos test | Walton, J. -04 (AC-joint pain) |
| IRRST; Internal Rotation Resistance Strength test | Zaslav, K. R. -01 (Discern internal vs external impingement) |

PETS-physical examination test of the shoulder, RCT-rotator cuff tear, SIS-subacromial impingement syndrome, SLAP-superior labrum anterior superior. **^1^**Significant errors were discovered in these articles, e.g. in construction of one or several 2X2 tables or there was confusion about which tests were reported in results, **^2^**Holtby et al.-04 in Arthroscopy, **^3^**Holtby et al.-04 in J Orthop Sports Phys Ther.

**Ver 1.2 – Date: 08.02.11**

**APPENDIX 2 QUADAS Scoring Manual**

**Quality Assessment tool* and Scoring Manual**

*Adapted from Whiting (2003), Cochrane Diagnostic Reviewers Handbook version 0.3 (2005) and a Cochrane protocol (Cochrane Library 2008, Issue 4, Physical tests for shoulder impingement syndrome, table 4)

Alternative 1:

**1. Was the spectrum of patients representative of the patients who will receive the test in practice?** [To define spectrum bias] Though clinical examination can be applied at all stages, our target population is the relatively selected one referred to a secondary or tertiary care (specialist) evaluation. This level of care may involve all kinds of specialist-centers which require referral as well as specialist evaluation in or in close collaboration with primary care that might represent a more unselected patient population.

**Y** (a) The setting was a specialist evaluation AND

(b) the population defined by age and gender AND

(c) there was diagnostic uncertainty (diagnosis wasn’t already established) AND

(d) the study was prospective AND

(e )recruitment was consecutive

**N** *General factors*

(a) There was no diagnostic uncertainty i.e. the study compared diseased with healthy subjects (case-control study) OR

(b) the study was not prospective OR

(c) recruitment was not consecutive

*Review-specific factors*

(a) The setting was not a specialist evaluation OR

(b) the population was clearly unselected OR self referred

**?** Insufficient information

**2. Were selection criteria clearly described?**

**Y** (**a**) The selection criteria were clearly described (e.g. pain in the shoulder/ deltoid region, painful arc of motion, pain on overhead activities contributing to a clinical suspicion of impingement) AND

(**b**) the exclusion criteria were clearly described (e.g. referred pain, gross restriction of movement, inflammatory disease, fracture)

**N** (**a**) The selection criteria were undescribed/very unclearly described (e.g. “shoulder pain”) OR (**b**) the exclusion criteria were undescribed/very unclearly described

**?** (**a**) The selection criteria were described AND

(**b**) the exclusion criteria were described BUT

(**c**) the description of the selection criteria was not completely clear (e.g. an unqualified statement such as, “patients with suspected impingement”) OR

(**d**) the description of the exclusion criteria was not completely clear

**3. Is the reference standard likely to correctly classify the target condition?**

Generally recognized ‘gold’ standards are not clearly defined for all the different conditions in the shoulder but there are exceptions. *Refer to separate table** for aid in the evaluation.*

**Y** Refer to separate table** for aid in the evaluation.

**N** Not applicable

**?** The reference standard was

(a) arthrography OR

(b) x-ray OR

(c) subacromial local anesthesia by ‘blind’ injection OR

(d) MRA, except as defined in separate table** OR

(e) MRI, except as defined in separate table** OR

(f) ultrasonography, except as defined in separate table** OR

(g) Other reference tests or tests used to classify other conditions than defined in the separate table** below.

**4. Is the time period between the reference standard and index test short enough to be reasonably sure that the target condition did not change between the two tests?** [To identify disease progression bias]

The acceptable interval would vary according to the average duration of symptoms.

**Y** The average interval was <

(a) the average duration of symptoms OR

(b) 1 month (whichever was the shorter)

**N** The conditions for ‘Y’ were expressly not met

**?** Insufficient information

**5. Did the whole sample, or a random selection of the sample, receive verification using a reference standard?** [To identify partial verification bias]

**Y** (a) All patients were accounted for as having undergone a reference test OR

(b) a randomly selected sample of patients underwent a reference test.

(Score ‘Y’ even if different reference tests were used)

**N** (a) Not all patients were accounted for as having undergone a reference test OR

(b) a non-random selection of patients underwent a reference test

**?** Insufficient information

**6. Did patients receive the same reference standard regardless of the index test result?** [To identify differential verification bias]

**Y** (a) All patients underwent the same reference test OR

(b) patients underwent different reference tests, but these were probably equivalent (e.g. arthroscopy and open surgery)

**N** Patients underwent different reference tests, which were probably not equivalent (e.g. arthrography and MR)

**?** Insufficient information

**7. Was the reference standard independent of the index test?** [To identify incorporation bias]

**Y** Self-explanatory

**N** Self-explanatory

**?** Self-explanatory

**8. Was the execution of the index test described in sufficient detail to permit replication of the test?**

**Y** (a) A clear, detailed description was given enabling replication and interpretation OR

(b) a reference was given to an adequate source of this information

**N** (a) The description lacked sufficient clarity to enable replication or interpretation AND

(b) no reference was given to an adequate source of this information

**?** Not applicable

**9. Was the execution of the reference standard described in sufficient detail to permit its replication?**

**Y** (a) A clear, detailed description was given enabling replication and interpretation OR

(b) a reference was given to an adequate source of this information

**N** (a) The description lacked sufficient clarity to enable replication or interpretation AND

(b) no reference was given to an adequate source of this information

**?** Not applicable

**10. Were the index test results interpreted without knowledge of the results of the reference standard?** [To identify test review bias?] Clinical examination is highly subjective, and retrospective interpretation is a potential concern.

**Y** There was a clear statement of blinding

**N** There does not appear to have been blinding

**?** The study was prospective and it is unclear whether there was blinding, but the index test preceded the reference standard. This does not apply to retrospective studies, in which both tests are likely to have been re-interpreted at the same time (Whiting 2003). In the absence of a clear statement of blinding, retrospective studies should be scored ‘N’

**11. Were the reference standard results interpreted without knowledge of the results of the index test?** [To identify diagnostic review bias] Since the clinical relevance of some arthroscopic and surgical findings (e.g. glenoid labral lesions, rotator cuff fraying and even rotator cuff tears) is uncertain, and interpretation of the other reference tests is subjective, foreknowledge of the index test result has potential to influence interpretation.

**Y** There was a clear statement of blinding

**N** There does not appear to have been blinding

**?** The reference test was stated to have been conducted “independently”

**12. Were the same clinical data available when test results were interpreted as would be available when the test is used in practice?** Patients’ demographic (age/ sex) and historical data would normally be available when physical test results are interpreted.

**Y** Demographic and historical data were available when index test/s was/were interpreted

**N** Demographic or historical data were not available when index test/s was/were interpreted

**?** Insufficient information

**13. Were uninterpretable/intermediate test results reported?**

**Y** (a) The study was prospective AND

(b) recruitment was consecutive AND

(c) test results were reported for all initially included patients

**N** (a) Recruitment was not consecutive OR

(b) test results were not reported for all initially included patients

**?** (a) Insufficient information OR

(b) the study was not prospective (due to inconsistent reporting in clinical records, uninterpretable/ intermediate test results are sometimes not identified in retrospective studies (van der Schouw 1995))

**14. Were withdrawals from the study explained?**

**Y** (a) The study was prospective AND

(b) recruitment was consecutive AND

(c) withdrawals were reported AND

(d) withdrawals were explained (ideally by a flow chart)

**N** (a) The study was not prospective OR

(b) recruitment was not consecutive (unexplained non-recruitment equating to unreported/explained withdrawal) OR

(c) withdrawals did not appear to have been reported OR

(d) withdrawals were unexplained

**?** Insufficient information

**** Aid for QUADAS question 3; Reference tests – definitions and list of adequate reference standards**

| Test | Definition | Adequate reference standard for: | Qualifications / comments |
| --- | --- | --- | --- |
| **Open surgery** | **A diagnostic ’gold’ standard.**  An invasive procedure during the course of which the interior of the shoulder joint and subacromial- subdeltoid bursa may be directly visualized through  an open incision. | (1) Subacromial impingement.  (2) Subacromial-subdeltoid  bursitis.  (3) Bursal side rotator cuff tears.  (4) Full thickness rotator cuff  tears.  (5) Biceps tendon pathology. | (1) Tears of the rotator cuff ’s internal  substance and joint side  may be missed, as may SLAP lesions and disorders of the long  head of biceps.  (2) Rotator cuff tears may be missed if obscured e.g. by inflammation. |
| **Arthroscopy** | **A diagnostic ’gold’ standard.**  A “keyhole” surgical procedure, in which the interior of the shoulder joint and subacromialsubdeltoid bursa may be visualized through a flexible fiber-optic tube. | (1) Subacromial-subdeltoid bursitis.  (2) Subacromial impingement.  (3) Antero-superior glenoid impingement.  (4) Postero-superior glenoid impingement.  (5) Bursal side rotator cuff tears.  (6) Full thick-ness rotator cuff tears.  (7) Joint side rotator cuff tears.  (8)Disorders of long head of biceps.  (9)Labral lesions, including SLAP | (1) There is a technical and interpretive learning curve.  (2) Tears of the rotator cuff ’s internal substance may be missed.  (3) Rotator cuff tears may be missed if obscured, e.g. by inflammation. |
| **Ultra-sonography** | A non-invasive diagnostic technique in which high-frequency sound waves are bounced (reflected) from the tissues in order to form images of the body’s internal structures. | (1) Full thickness rotator cuff tears. | (1) Technique and inter-pretation are highly operator-dependent. The presence/absence of data/material confirming accuracy in individual diagnostic studies should be taken into account.  (2) SLAP lesions cannot be visualized  using ultrasound. |
| **Magnetic Resonance Imaging (MRI)** | A non-invasive diagnostic technique. Tissues’ differing responses in a strong electromagnetic field are analyzed by computer and translated into an accurate anatomical image. | (1) Full thickness rotator cuff tears. | This applies in settings (such  as general primary care) where  there is likely to be a low incidence  of this disorder. |
| **Arthrography** | A diagnostic technique  in which X-rays are taken after injection of a fluid contrast material into a joint. | (1) Joint side rotator cuff tears.  (2) Full thickness rotator cuff tears. |  |
| **Magnetic Resonance Arthrography(MRA)** | A combination of Magnetic Resonance Imaging (MRI) and arthrography. An MRI scan is done after injection of contrast material into a joint. | (1) Joint side rotator cuff tears.  (2) Full thickness rotator cuff  tears.  (3) SLAP (and labral) lesions.  (4) Biceps pathology. | 1) Saline as sole contrast agent may be less efficient for diagnosing rotator cuff tears than combination with eg gadolinum |
| **Bursography** | A diagnostic technique  in which X-rays are taken after injection of a contrast material into a bursa. | (1) Bursal side rotator cuff tears. |  |
| **Local anaesthesia** | A minimally invasive procedure in which a local anesthetic is injected and the effect on signs and/or symptoms noted. | (1) Subacromial outlet impingement.  (2) AC joint | (1) Correct interpretation is dependent on the injection’s accuracy.  2) Cuff tears may cause ‘leak’ between bursa and joint |
| **X-ray** | Ordinary X-ray | (1) Osteo-arthritis of the Glenohumeral and AC joint |  |

**APPENDIX 3:** Version 2.0 Date: 19.10.2010

ELIGIBILITY CRITERIA FOR INCLUSION OF ARTICLES(Abstracts 1-5 only):

1. At least one physical examination test of the shoulder girdle is studied OR diagnostic injection of local anesthetics in the shoulder, AC-joint, sternoclavicular or thoracoscapular joint is studied.
2. Tests are compared with a criterion standard.
3. Studies that only regard tests of fractures and dislocations of joints as well as tests that only regard nerve dysfunction are excluded.
4. Living humans are studied (Exclusion of studies where tests are only performed on animals or cadavers as well as tests performed under general anesthesia)
5. Article is in English or Scandinavian languages (Swedish, Danish and Norwegian)
6. Number of individuals included in study is at least 20
7. One of the paired statistics of sensitivity and specificity are reported or can be discerned for an individual test.
8. One of the following may be used as criterion standard for:
   1. Impingement
      1. ARTHROSCOPY / SURGERY
      2. MRI OR MR-ARTHROGRAPHY
      3. ULTRASOUND
      4. ULTRASOUND/ FLUOROSCOPY GUIDED INJECTION OF LOCAL ANESTHETIC
      5. “BLIND” INJECTION OF LOCAL ANESTHETIC
   2. AC-joint
      1. ULTRASOUND GUIDED INJECTION OF LOCAL ANESTHETIC
      2. INJECTION OF LOCAL ANESTHETIC
      3. MRI OR MRI-ARTHROGRAPHY
      4. ULTRASOUND
      5. ARTHROSCOPY / SURGERY
      6. X-ray
   3. Rupture of the rotator cuff
      1. ARTHROSCOPY / SURGERY
      2. MRI OR MRI-ARTHROGRAPHY
      3. ULTRASOUND
      4. Arthrograpy
      5. Bursography
   4. Biceps inflammation / rupture
      1. MRI OR MRI-ARTHROGRAPHY
      2. ARTHROSCOPY / SURGERY
      3. ULTRASOUND
      4. ULTRASOUND GUIDED INJECTION OF LOCAL ANESTHETIC
      5. INJECTION OF LOCAL ANESTHETIC
   5. Glenohumeral instability
      1. ARTHROSCOPY / SURGERY
      2. MRI OR MRI ARTHROGRAPHY
   6. Adhesive capsulitis
      1. MRI OR MRI-ARTHROGRAPHY
      2. ARTHROSCOPY / SURGERY
   7. Glenohumeral osteoarthritis
      1. MRI OR MRI-ARTHROGRAPHY
      2. X-ray
      3. ARTHROSCOPY / SURGERY
   8. SLAP-lesions
      1. ARTHROSCOPY / SURGERY
      2. MRI OR MR-ARTHROGRAPHY

Systematic reviews are excluded but the reference list will be browsed for eligible articles.
